# Hypoxia modulates the antioxidant effect of hydroxytyrosol in MCF-7 breast cancer cells

**DOI:** 10.1371/journal.pone.0203892

**Published:** 2018-09-20

**Authors:** Jesús Calahorra, Esther Martínez-Lara, Cristina De Dios, Eva Siles

**Affiliations:** Department of Experimental Biology, University of Jaén, Campus Las Lagunillas s/n, Jaén, Spain; University of Nebraska Medical Center, UNITED STATES

## Abstract

Although cancer is multifactorial, a strong correlation between this pathology and increased oxidative stress has long been stablished. Hypoxia, inherent to solid tumors, increases reactive oxygen species and should be taken into account when analyzing the response of tumor cells to antioxidants. The Mediterranean diet has been related to a lower incidence of cancer, and particularly of breast cancer. Given that hydroxytyrosol (HT) is largely responsible for the antioxidant properties of olive oil, we have performed a comprehensive and comparative study of its effect on the oxidative stress response of the human breast cancer cell line MCF-7 in hypoxia and normoxia. Our results demonstrate that the antioxidant action of HT is particularly effective in a hypoxic environment. Moreover, we have observed that this polyphenol modulates the transcription and translation of members of the PGC-1α/ERRα and PGC-1α/Nrf2 pathways. However, while the transcriptional effects of HT are similar in normoxic and hypoxic conditions, its translational action is less prominent and partially attenuated in hypoxia, and therefore cannot completely explain the antioxidant effect of HT. Consequently, our results underscore that the hypoxic environment of tumor cells should be considered when analyzing the effect of bioactive compounds. Besides, this study also points to the importance of assessing the regulatory role of HT at both mRNA and protein level to get a complete picture of its effects.

## Introduction

Cancer is a generic term for a large group of diseases which figure among the leading causes of morbidity and mortality worldwide. In women, the most common cancer is breast cancer, contributing more than 25% of the total number of new cases diagnosed in 2012 [1]. Although cancer is multifactorial, a strong correlation between this pathology and increased oxidative stress has long been stablished. In fact, overproduction of reactive oxygen species (ROS) as a consequence of genetic, metabolic and microenvironment-associated alterations is known to promote both tumor initiation and progression [2].

Oxidative stress is the result of an unbalance between ROS production and detoxification. This species are constantly formed in aerobic organisms, particularly in mitochondria. In this sense, although cancer cells show high rates of glycolysis, they also retain functional mitochondria, essential for tumorigenesis, in which oxidative phosphorylation occurs [3]. Mitochondrial biogenesis is regulated by PPARγ coactivator-1α (PGC-1α). This protein interacts and coactivates the estrogen-related receptor α (ERRα) and the nuclear respiratory factor 2 (Nrf2) [4]. ERRα is an “orphan” nuclear receptor whose activity is not regulated by ligand binding. This protein induces the transcription of the enzymes of the tricarboxylic acid cycle and oxidative phosphorylation, enabling adaptive metabolic events, such as biomass synthesis, that are crucial in cancer development [5]. Nrf2 acts as a heterodimer with other transcription factors such as Mafs, promoting the expression of target genes with one or more antioxidant response elements (ARE, TCAG/CXXXGC) in their promoter region. Among others, those targets are antioxidant, NADPH generating and glutathione synthesis enzymes, proteins involved in xenobiotic metabolism and efflux, and heat shock proteins [6]. The central function of ERRα and Nrf2 reinforces the importance of finding new molecules that modulates their activity, particularly under pathological situations highly linked to oxidative stress such as cancer.

In solid tumors, the high cell proliferation, the abnormal anatomy of blood vessels and their obstruction or compression imply the appearance of hypoxic areas. As an example, the average O_2_ pressure in breast tumors is approximately 80% lower than in normal breast tissue [7]. This hypoxic microenvironment further increases oxidative stress, promoting tumor development and malignancy, and should be taken into account when performing studies about the molecular response of tumor cells.

A number of studies have revealed that in the Mediterranean countries the incidence of breast cancer is lower than in other developed countries, pointing to the dietary practice as possible cause of this effect [8–10]. The Mediterranean diet is characterized by a high intake of fruits, vegetables, whole grains, legumes and healthy fats such as olive oil. Olive oil is mainly composed of fatty acids, particularly oleic acid and linoleic acid. However, in recent years much attention has been paid to the minor components of olive oil. This fraction represents no more than 2% of the olive oil weight and includes flavonoids, lignans or secoiridoids and phenolic compounds such as hydroxytyrosol (3,4-dihydroxyphenylethanol; HT), endowed with antioxidant properties [11]. Although some authors have studied the antioxidant action of this polyphenol in breast cancer [12–14], there is a lack of a comprehensive analysis of its molecular effect in hypoxic conditions. With this background, and considering the importance of hypoxia in tumor microenvironment, the objective of the present study was to comparatively analyze the effect of HT treatment in the oxidative response of hypoxic and normoxic MCF-7 cells, widely used in breast cancer research.

## Materials and methods

### Chemicals and reagents

HT (purity ≥98%) was obtained from Extrasynthese (Lyon, France). Dulbecco's modified Eagle's medium (DMEM) was from BiochromAG (Berlin, Germany). RNA was isolated using the RNeasyPlus Mini kit (Qiagen, Hilden, Germany) and cDNA was prepared with Maxima First Strand cDNA Synthesis Kit for RT-qPCR (Fermentas Intl., Vilnius, Lithuania). Real-time PCR was performed using SYBR Fast Master Mix (2x) Universal (KAPABiosystems, Massachusetts, USA). Primers were synthesized by Biomedal S.L. (Sevilla, Spain). Nrf2 siRNA (sc-37049), scramble siRNA (scr siRNA) and the transfection reagent were from Santa Cruz Biotechnology (CA, USA). Opti-MEM reduced serum medium was bought from Thermo Fisher Scientific. α-Tubulin antibody, foetal bovine serum (FBS), sulforhodamine B (SRB), trichloroacetic acid (TCA), 2´,7´-dichlorofluorescin diacetate (DCFH-DA), glutathione reductase (GR), reduced glutathione, NADPH, cumene hydroperoxide, and other general reagents were from Sigma (St. Louis, MO, USA). Heme oxygenase-1 (HO-1) was measured with the enzyme linked immunosorbent assay (ELISA) kit CSB-E08266h from Cusabio (Barksdale, USA). Primary antibodies (PGC-1α, Nrf2, ERRα, SIRT3) were purchased from Santa Cruz Biotechnology, Inc., (CA, USA) except anti-α-tubulin (Sigma, St. Louis, Mo, USA).

### Cell culture and treatments

Human breast cancer MCF-7 cells were grown in 10% foetal bovine serum supplemented DMEM at 37°C in 5% CO_2_ and 21% O_2_. Cells were pre-treated or not with HT, dissolved in ethanol immediately before use, for 16 h under normoxic conditions (21% O_2_), being cultured during the last 4 h either in normoxic or hypoxic conditions (1% O_2_). Controls were treated with an equal ethanol concentration.

### Cytotoxicity assay

The cytotoxic effect of HT was evaluated using the SRB assay as previously described [15]. Briefly, 4 x 10^4^ cells/well were plated in 24-well tissue culture plates (Nunc, Rosbilde, Denmark). The plates were incubated for 24 h to allow the cells to adhere. HT was then added to the corresponding wells at a range of concentrations (0 to 600 μM), each concentration being used in at least four replicate wells. After 16 h of treatment, last 4 h either in normoxic or hypoxic conditions, the medium was removed and the cultures were washed with PBS. Cells were fixed at 4°C with 10% TCA for 30 min and then washed with tap water to remove TCA. Plates were air dried and stored until use. TCA-fixed cells were stained for 20 min with 0.4% (w/v) SRB dissolved in 1% acetic acid. After staining, SRB was removed and cultures were rinsed with 1% acetic acid to eliminate unbound dye. The cultures were air dried and bound dye was solubilized with 10 mM Tris base (pH 10.5). Optical density was read in a plate reader (ThermoLabSystem Multiscan Ascent) at 492 nm. Cell survival was measured as the percentage of absorbance compared with that obtained in non HT-treated cells.

### Measurement of intracellular generation of ROS

Intracellular generation of ROS was analysed using DCFH-DA as a probe [16]. ROS in the cells oxidize DCFH, yielding highly fluorescent 2´,7´-dichlorofluorescein (DCF). Briefly, cells were cultured in 96-well plates (10^4^ cells/well) and treated for 16 h with HT (0–200μM), being cultured during the last 4 h either in normoxic or hypoxic conditions. 30 min before the end of the experiment, cells were washed with Krebs buffer (pH 7.3) and incubated with 10 μM DCFH-DA. Once the incubation was finished, cells were washed three times and DCF fluorescence was measured in a plate reader (ThermoLabSystem Multiscan Ascent) at an excitation wavelength of 488 nm and emission wavelength of 535 nm.

### Quantitative Real-time PCR (qRT-PCR)

Gene expression of PGC-1α, ERRα, Nrf2, HO-1, GSTA2 (Glutathione S-Transferase Alpha 2), and SIRT3 (Sirtuin-3) were quantitatively assessed by real-time PCR using peptidylprolylisomerase A (PPIA) as the normalizing gene. Real-time PCR was performed in a MxPro thermal cycler (Stratagene, California, USA) using SYBR Fast Master Mix (2x) Universal. The sequences of primers are shown in Table 1. Experiments were performed in triplicate, and the relative quantities of target genes, corrected with the normalizing gene PPIA, were calculated using the Stratagene MxProTM QPCR Software.

**Table 1:** Primer details.

primer	forward (5´-3´)	reverse (5´-3´)
PGC-1α	TGCTTTTGCTGCTCTTGAAA	TTACCTGCGCAAGCTTCTCT
ERRα	GCTGCCCTGCTGCAACTA	GCCTCGTGCAGAGCTTCTC
Nrf2	TCAGGCTCAGTCACCTGAAA	TTGGCTTCTGGACTTGGAAC
HO-1	ATGACACCAAGGACCAGAGC	GTGTAAGGACCCATCGGAGA
GSTA2	GAGCCACGGACAAGACTACC	CACTGTGGGCAGGTTACTGA
SIRT3	AACATCGATGGGCTTGAGAG	AGAACACAATGTCGGGCTTC
PPIA	TTCATCTGCACTGCCAAGAC	TCGAGTTGTCCACAGTCAGC

### Western blot

For western blot analysis, equal amounts of denatured total-protein extracts (30 μg) were loaded and separated on a 7.5% (PGC-1α), or 10% (SIRT3, Nrf2 and ERRα) SDS-polyacrylamide gel. Proteins in the gel were transferred to a PVDF membrane and then blocked. Monoclonal antibodies to PGC-1α, Nrf2, ERRα, SIRT3 and to α-tubulin, as a loading control, were used for detection of the respective proteins. Antibody reaction was revealed by means of chemiluminescence detection procedures according to the manufacturer's recommendations (ECL kit, Amersham Corp., Buckinghamshire, UK).

### Se-independent glutathione peroxidase activity

Se-independent glutathione peroxidase (GPX) activity, as indicative of GSTA2 activity, was determined in a coupled assay with GR using cumene hydroperoxide as a substrate [17]. To prepare samples, at the end of each incubation period, cells were collected, washed with cold PBS, lysed for 20 min at 4°C in EBC buffer (20 mM Tris–HCl pH 8; 150 mM NaCl, 1 mM EDTA, 0.5% NP-40) and sonicated. After centrifugation at 14,000 *g* for 15 min at 4°C, supernatants were collected and protein was quantified [18].

### HO-1 determination

HO-1 was measured with a commercial ELISA kit according to manufacturer's instructions. To prepare samples, cells of each experimental condition were collected and lysed in PBS by sonication. After centrifugation at 14,000 *g* for 30 min, supernatants were separated and protein was quantified [18].

### Nrf2 siRNA transfection

Nrf2 siRNA and scr siRNA were transfected with transfection reagent according to the protocol of the manufacturer´s. Briefly, MCF-7 cells (16 × 10^4^/well) were plated into 6-well plates, allowed to adhere for 24 h and incubated with fresh Opti-MEM Reduced Serum Medium containing siNrf2 (50 nM) or scr-siRNA (50 nM) for 5 h. The transfection medium was then replaced with fresh DMEM containing 10% FBS for 48 h before further treatment with HT and/or hypoxia. To quantify the efficiency of siNrf2, siNrf2 and scr siRNA transfected MCF-7 cells were lysed and prepared for determining Nrf2 mRNA expression by RT-PCR.

### Statistical analysis

Data are expressed as means ± SD of at least three independent experiments. Statistical comparisons between the different experimental groups and their corresponding controls were made with Student’s t-test, accepting *p<*0.05 as the level of significance, using GraphPad Prism 6 software (GraphPad Software Inc.).

## Results

### Cytotoxic effect of HT on MCF-7 cells

**Fig 1:**
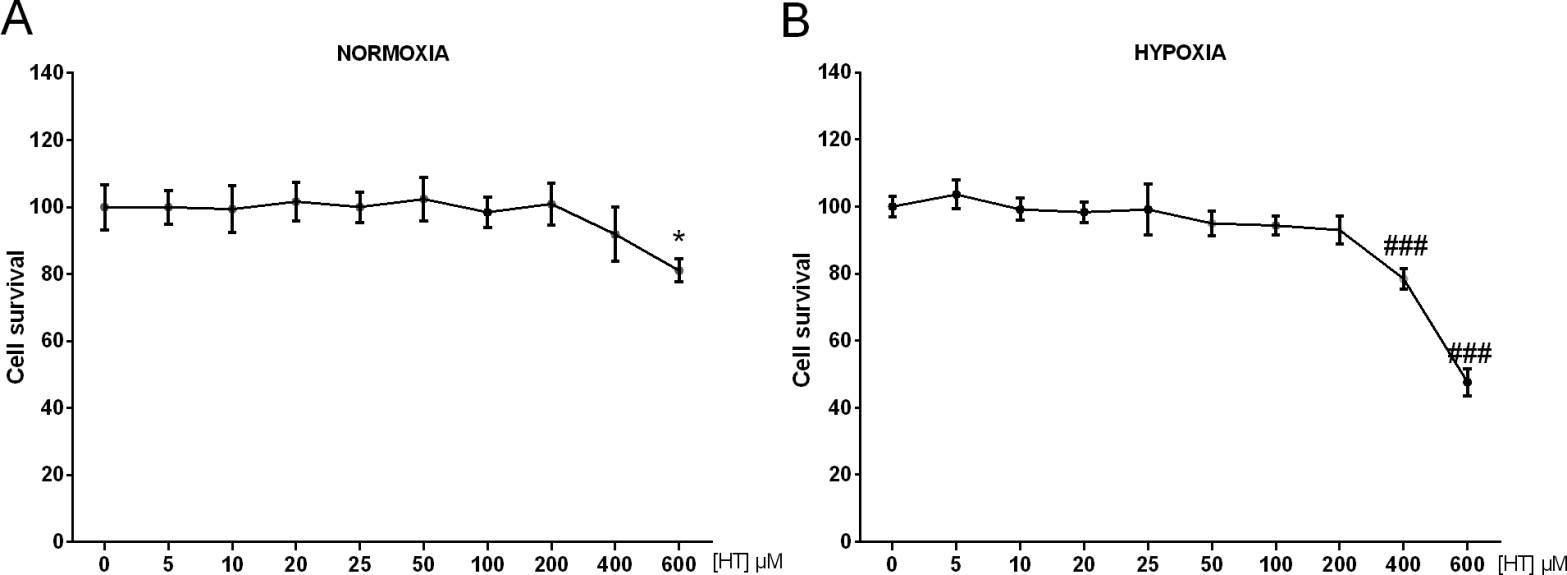
High concentrations of HT are necessary to affect MCF-7 cell proliferation. The cytotoxic effect of HT was evaluated in (A) normoxic cells and (B) hypoxic cells by the SRB assay. Values represent the mean ± SD from three independent experiments. Statistically significant differences with the corresponding non-treated normoxic or hypoxic cells:* p<0.05 and ^###^ p<0.001, respectively.

The cytotoxic effect of HT in MCF-7 cells after 16 h of treatment is shown in Fig 1A. Our results indicated that concentrations as high as 600 μM were necessary to affect cell proliferation. To comparatively analyse the effect of HT in normoxic and hypoxic conditions, we evaluated whether the cytotoxicity of HT could be influenced by a decrease in O_2_ pressure. To achieve this goal, MCF-7 cells were incubated under hypoxia during the last 4 h of HT-treatment. This experimental condition has been previously used by our group [19] and is strong enough to achieve an adaptive response to this situation, such as the induction of hypoxia inducible factor-1 (results not shown). As shown in Fig 1B, a 400 μM HT-treatment resulted toxic to MCF-7 cells, suggesting that hypoxia increases HT cytotoxicity although both, 400 and 600 μM, are extremely high concentrations.

### HT exerts an antioxidant effect in hypoxic conditions

**Fig 2:**
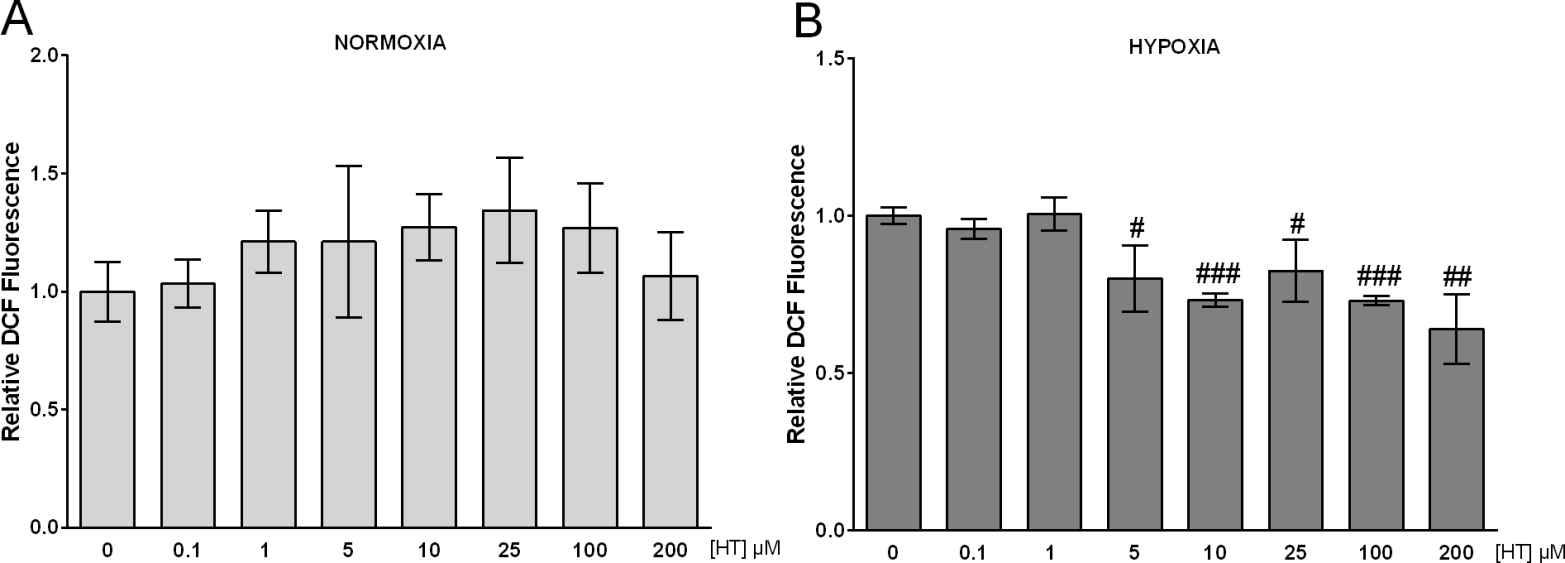
HT decreases the oxidative stress level only in hypoxic conditions. Oxidative stress level measured by DCF fluorescence in (A) normoxic cells and (B) hypoxic cells. Values represent the mean ± SD from three independent experiments. Statistically significant differences with the corresponding non-treated hypoxic cells: ^#^ p<0.05, ^##^ p<0.01 and ^###^ p<0.001.

HT is reportedly described as an antioxidant compound. Thus, we next evaluated the effect of the treatment of MCF-7 cells with sub-cytotoxic concentrations of HT (0.1–200 μM) on the oxidative stress level. In normoxia, none of the concentrations assayed exerted any effect on this parameter (Fig 2A). However, when we analysed those same concentrations in hypoxic conditions (Fig 2B), a significant decrease was achieved by HT 5 μM and persisted until 200 μM.

### PGC-1α expression is differentially regulated by HT in hypoxia

**Fig 3:**
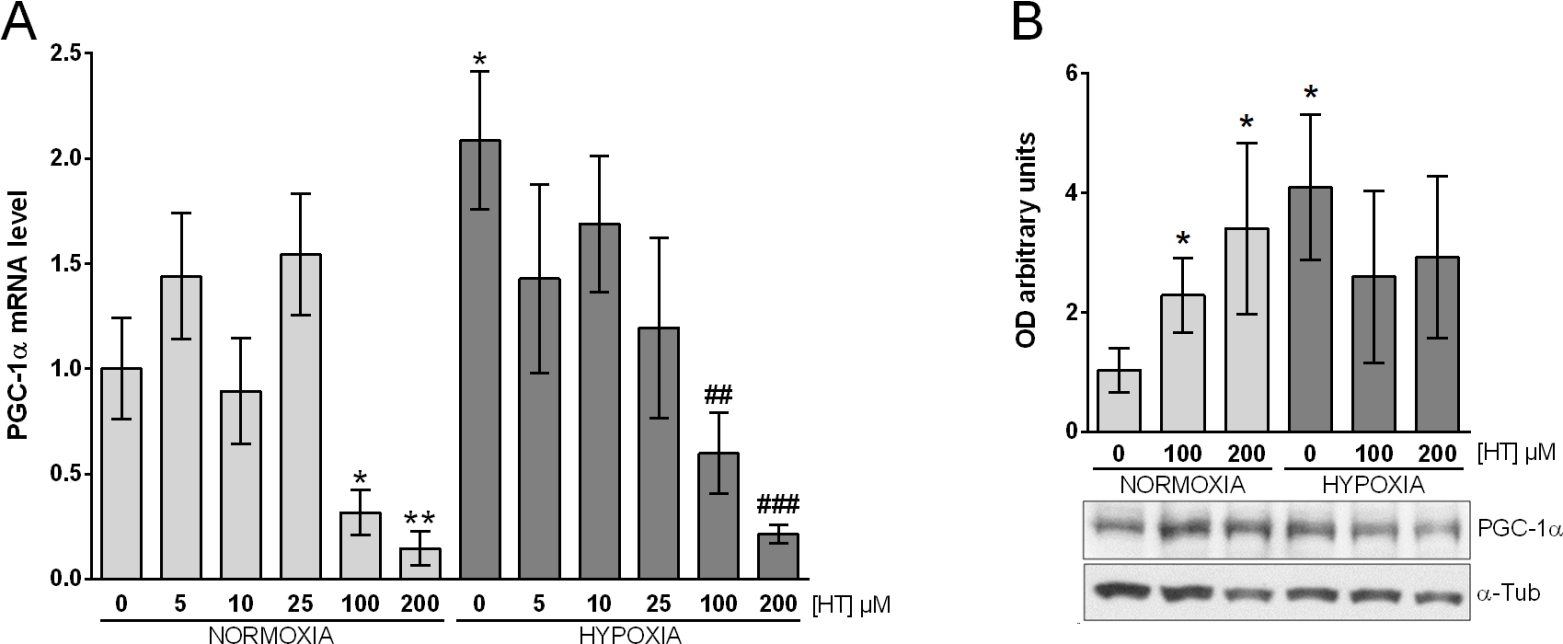
PGC-1α expression is differentially regulated by HT in normoxic and hypoxic conditions. (A) PGC-1α mRNA levels relative to normoxic non HT-treated cells after normalization against PPIA. (B) Densitometric quantifications of PGC-1α protein relative to α-tubulin (α-Tub). A representative immunoblot from a single experiment is shown. Values represent the mean ± SD from three independent experiments. Statistically significant differences with the corresponding non-treated normoxic cells: * p<0.05, ** *p*<0.01. Statistically significant differences with the corresponding non-treated hypoxic cells: ^##^ *p*<0.01, ^###^ *p*<0.001.

Given the impact of HT on the oxidative stress level, we evaluated whether this effect was related to a change in PGC-1α expression (Fig 3). The analysis of the transcriptional level of PGC-1α (Fig 3A) indicated that, although HT exerted no effect on the transcription of this coactivator at low concentrations, it decreased its level at high doses (100 and 200 μM), both in normoxic and in hypoxic conditions. This result prompted us to determine whether PGC-1α protein was also down-regulated at 100 and 200μM. As shown in Fig 3B, the transcriptional changes induced by HT did not parallel the expression of PGC-1α at the protein level. In normoxic conditions, the same doses of HT that decreased transcription promoted the upregulation of the PGC-1α protein, while HT seemed to exert no effect in a hypoxic environment. Hence, the transcriptional and translational effects of HT do not seem to follow a similar pattern of response.

### ERRα expression is hardly affected by HT independently of the O_2_ pressure

**Fig 4:**
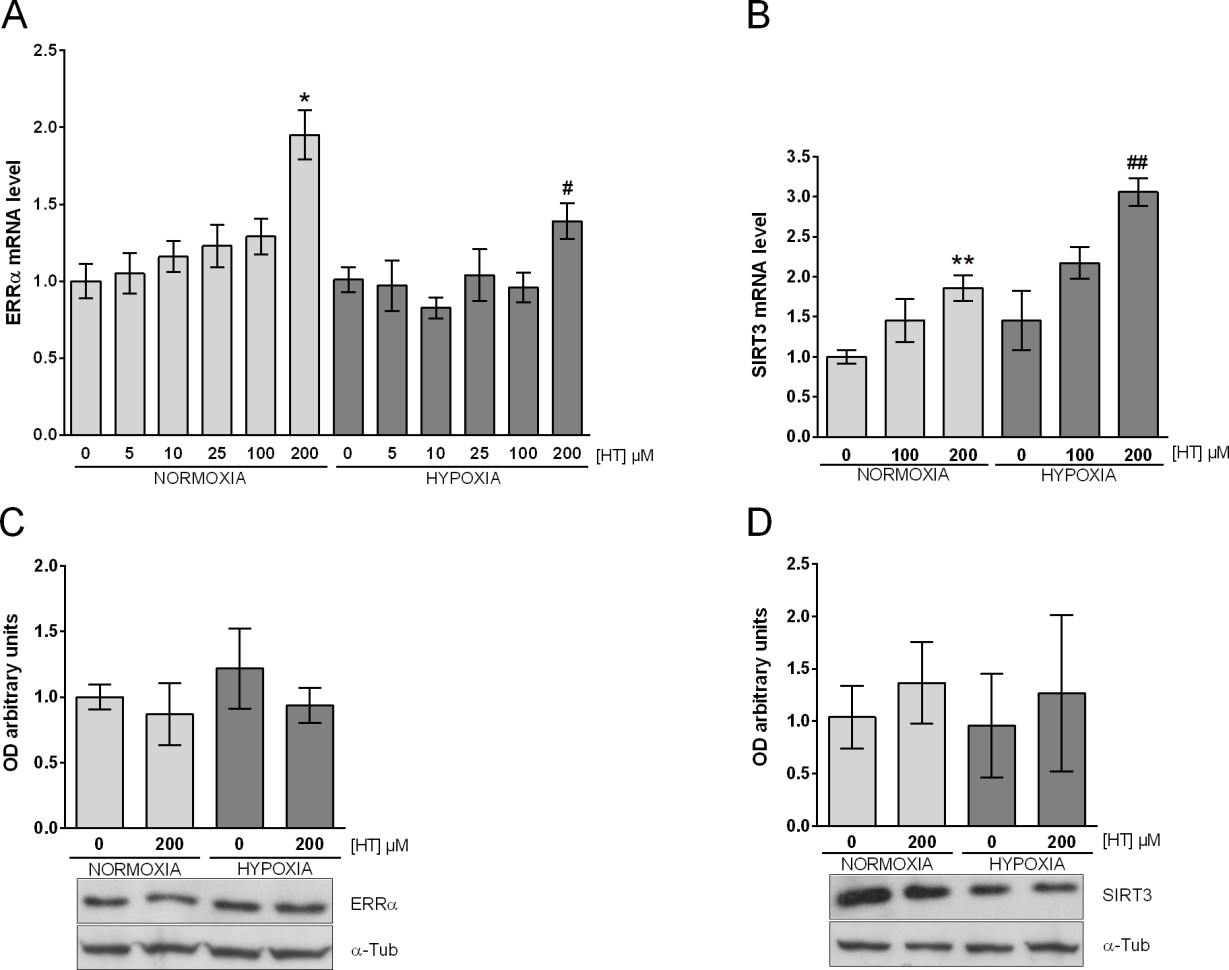
HT exerts a similar effect on the PGC-1α/ERRα pathway in normoxia and hypoxia. (A) ERRα and (B) SIRT3 mRNA levels. The results are expressed as mRNA expression relative to normoxic non HT-treated cells after normalization against PPIA. Densitometric quantifications of (C) ERRα and (D) SIRT3 protein relative to α-tubulin (α-Tub). A representative immunoblot from a single experiment is shown. Values represent the mean ± SD from three independent experiments. Statistically significant differences with the corresponding non-treated normoxic cells: * p<0.05, ** *p*<0.01. Statistically significant differences with the corresponding non-treated hypoxic cells: ^#^ p<0.05, ^##^ *p*<0.01.

The effect of PGC-1α is mediated by coactivation of transcription factors such as ERRα. The differential response of PGC-1α to HT treatment in normoxic and hypoxic conditions led us to evaluate the influence of this polyphenol on ERRα levels. Our results (Fig 4A) indicated that, the ERRα mRNA expression was not affected either by hypoxia or by HT-treatment with the exception of the 200 μM HT-dose, which induced ERRα levels independently of the oxygen concentration. However, no changes were observed when we analysed the expression of ERRα at the protein level (Fig 4C). After confirming that HT exerted a different regulation on PGC-1α and ERRα at 100 and 200 μM, we addressed the activity of the PGC-1α/ERRα pathway at both doses by evaluating the expression of one of its target gene, SIRT3. As shown in Fig 4B, SIRT3 mRNA expression was only increased by a 200 μM HT-treatment in normoxia and hypoxia. However, again this increase did not lead to higher protein levels (Fig 4D). Consequently, it may be assumed that although HT transcriptionally regulates the PGC-1α/ERRα pathway, this effect is not finally reflected at the protein level.

### HT consistently up-regulates Nrf2 in normoxia and hypoxia

**Fig 5:**
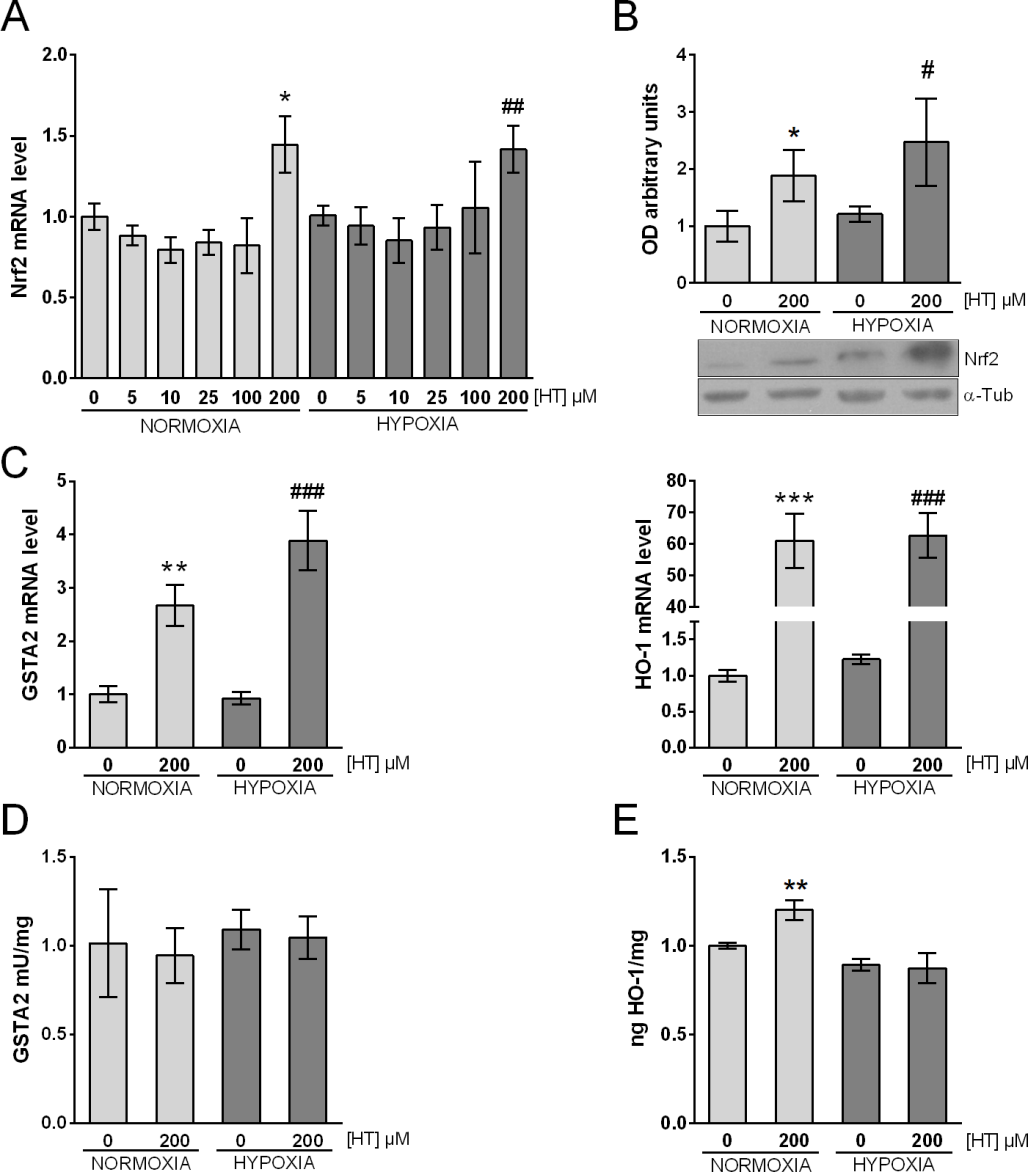
HT up-regulates Nrf2 both in normoxic and hypoxic conditions. (A) Transcriptional levels of Nrf2 in HT-treated (0–200 μM) normoxic and hypoxic cells. Effect of HT 200 μM on: (B) Nrf2 protein expression, (C, D) mRNA levels of the Nrf2 target genes GSTA2 and HO-1, (E) GSTA2 activity, and (F) HO-1 protein level. The mRNA results are expressed as relative to normoxic non HT-treated cells after normalization against PPIA. Densitometric quantification of Nrf2 protein is shown as relative to α-tubulin (α-Tub). A representative immunoblot from a single experiment is shown. Values represent the mean ± SD from three independent experiments. Statistically significant differences with the corresponding non-treated normoxic cells: * p<0.05, ** p<0.01, *** p<0.001. Statistically significant differences with the corresponding non-treated hypoxic cells: ^#^ p<0.05, ^##^ p<0.01, ^###^ p<0.001.

In addition to analysing the expression and activity of the PGC-1α/ERRα pathway, we also evaluated the effect of HT on Nrf2 (Fig 5). As shown in Fig 5A, the transcription of Nrf2 increased at the highest concentration analysed (200 μM), both in normoxia and hypoxia. In this case, however, the transcriptional upregulation was accompanied by a parallel protein increase (Fig 5B). Having demonstrated the effect of HT 200 μM on Nrf2, we monitored the activity of this transcription factor by quantifying the mRNA expression of GSTA2 and HO-1, two antioxidant proteins induced by Nrf2. As shown in Fig 5C and 5D, GSTA2 and HO-1 mRNA levels were increased at 200 μM, both in normoxia and hypoxia, perfectly paralleling Nrf2 induction. To our knowledge, no GSTA2-antibodies are commercialized. However, GSTA2 exhibits a Se-independent GPX activity that can be evaluated by using cumene hydroperoxide as a substrate [20]. The quantitation of this activity in the different experimental conditions indicated that neither hypoxia nor HT-treatment promoted any change (Fig 5E). However, HT induced the expression of HO-1 (Fig 5F) although this effect was only achieved in normoxic conditions.

### HT induces HO-1 independently of Nrf2

**Fig 6:**
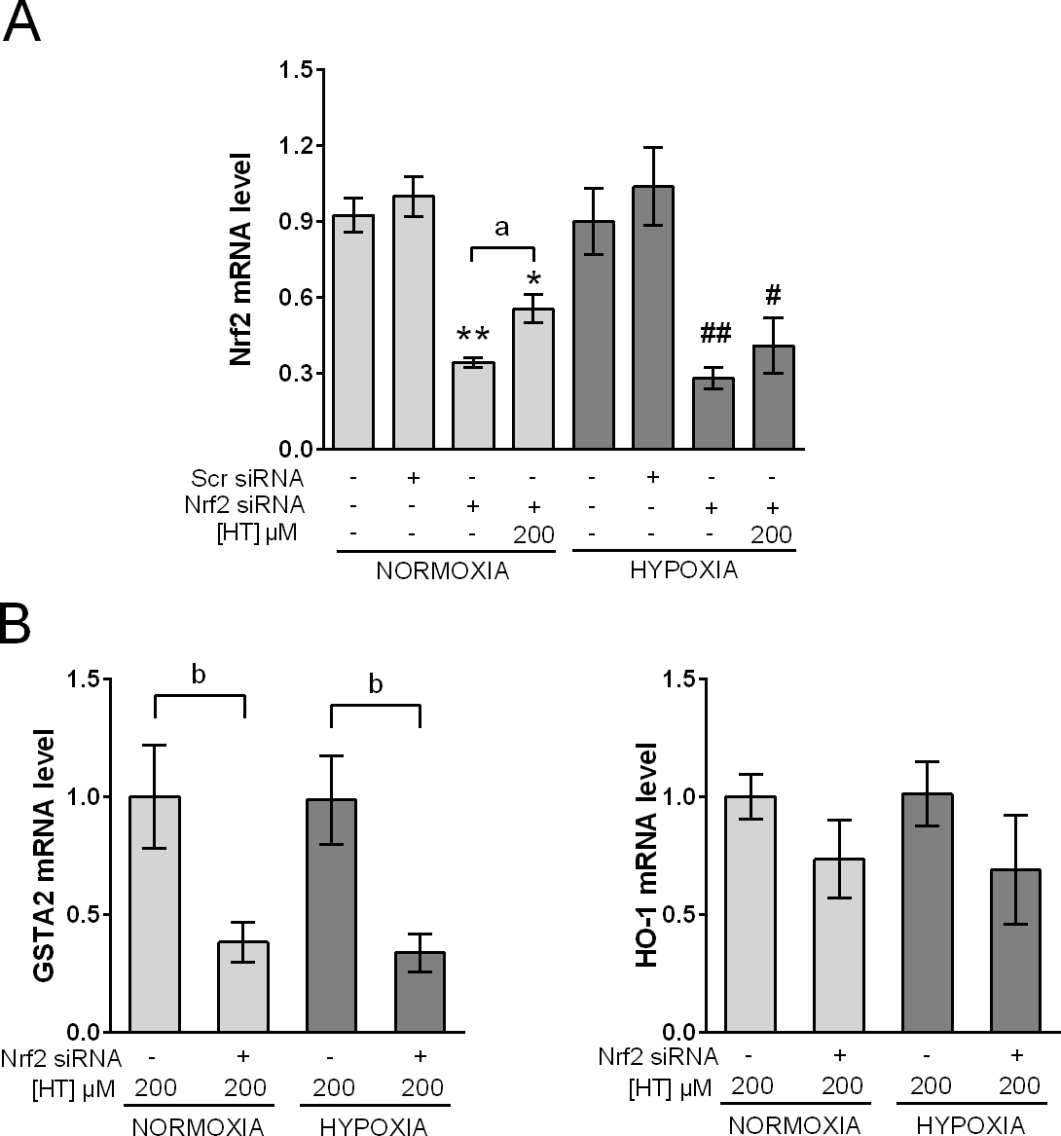
Nrf2 silencing reduces GSTA2 but not HO-1 mRNA levels. (A) Knockdown of Nrf2 with siNrf2 in control and HT-treated (200 μM) normoxic and hypoxic cells. The results are expressed as mRNA level relative to normoxic scr siRNA/non HT-treated cells after normalization against PPIA. (B) Effect of Nrf2-silencing on the transcription level of GSTA2 and HO-1 in normoxic and hypoxic cells after treatment with HT 200 μM. The results are expressed as mRNA levels relative to normoxic HT-treated cells after normalization against PPIA. Values represent the mean ± SD from three independent experiments. Statistically significant differences with the corresponding non-treated normoxic cells: * p<0.05, ** p<0.01. Statistically significant differences with the corresponding non-treated hypoxic cells: ^#^ p<0.05, ^##^ p<0.01. Statistically significant differences with the corresponding Nrf2-silenced non-HT-treated cells: ^a^ p<0.05. Statistically significant differences with the corresponding non-Nrf2-silenced HT-treated cells: ^b^ p<0.05.

To further determine the responsibility of Nrf2 on the increased transcription of GSTA2 and HO-1 in cells treated with 200 μM HT, we determined their mRNA levels after knocking-down Nrf2 by siRNA (Fig 6A). Our results (Fig 6B) indicated that, in these conditions, the expression of GSTA2 was significantly reduced, suggesting that Nrf2 is largely responsible for its transcriptional induction both in normoxia and in hypoxia. Nevertheless, HO-1 mRNA levels were not significantly affected by Nrf2 silencing. Consequently, it may be assumed that the huge response in the transcription of this enzyme to HT is not exclusively linked to Nrf2.

## Discussion

The intake of food rich in bioactive components such as polyphenols has been related to a lower incidence of tumors, and particularly to breast cancer [8–10]. In an *in vitro* study with human embryonic kidney renal cells, we previously demonstrated that HT, a phenolic compound considered highly responsible for the beneficial effects of olive oil, modulates the response to hypoxia [19]. Hypoxia is crucial in tumor microenvironment and in the response to anti-cancer treatments. Although in recent years much effort has been dedicated to understand the molecular mechanisms underlying the effect of HT in cancer cells, and particularly in breast cancer cells, there is a lack of a comprehensive study of the way hypoxia modulates its action. Breast cancer is often modelled using established cell lines. However, breast cancer is a remarkable heterogeneous disease that can be classified into different subtypes with diverse prognosis and response to treatments. MCF-7 cells, one of the most commonly used breast cancer cell line in the world, have been extensively employed to assess the antioxidant effect of HT. Therefore, in the present study we use these cells, representative of luminal A breast cancer, to assess the influence of hypoxia in the antioxidant response to HT.

The effect of HT on the proliferation rate of MCF-7 cells has been previously reported in the literature. Although the data may differ according to the particular experimental conditions, there is strong evidence in support of the concept that high concentrations of HT are necessary to achieve a cytotoxic effect on this cell line. Particularly, Han et al. [21] described that an incubation with 50 μg/ml of HT (~324 μM) induced apoptosis in MCF-7 cells after 12 h of treatment. Warletta et al. [12] was unable to report any effect on the cell proliferation rate when this cell line was treated for 24 h with up to 100 μM of HT. More recently, El-azem et al. [14] described that, when cells were treated for 72 h with HT, 50 μM was the minimum concentration able to achieve a significant but low decrease on cell proliferation. Our results, came to the same conclusion as concentrations as high as 600 μM were necessary to affect cell proliferation when we treated cells with HT for 16 h. However, the main aim of this work is to comparatively analyse the effect of HT in normoxic and hypoxic conditions. Therefore, we evaluated if the cytotoxicity of HT could be influenced by a decrease in the O_2_ pressure. In those conditions, a treatment with HT 400 μM results toxic, supporting, for the first time, the proposition that hypoxia increases HT cytotoxicity. Phenolic concentration in extra-virgen olive oil (EVOO) depends on several variables: (i) the olive cultivar and the ripening stage of fruit; (ii) environmental factors; (iii) extraction conditions and systems; and (iv) storage conditions and time [22]. Therefore, the data regarding the particular values of HT in EVOO are very heterogeneous. Some authors point that, at best, the content of HT can reach levels of about 7.5 mg/Kg [23]. However, other studies point to values around 60 mg/Kg [24]. Similar discordances are observed if we focus on the plasma concentration of HT after the ingestion of EVOO. If the degree of absorption proposed by Visioli et al. [25] is considered, up to 49 μM hydroxytyrosol could be found in plasma [26]. Nevertheless, it has been also published [27] that the maximum plasma concentration of free HT after an ingestion of 25 ml of EVOO reaches 4.4 ng/mL (approximately 28 nM). In any case, those concentrations are far from the ones we and others have described as cytotoxic. Hence, although the susceptibility to HT may differ according to the cancer cell line, it seems that the concentration of HT in EVOO is not likely to exert a toxic effect on breast cancer cells either in normoxic or in hypoxic conditions. However, the additive effect of the many other bioactive compounds of olive oil cannot be dismissed.

Cancer cells, due to altered metabolism, inadequate tumor vascular network and macrophage infiltration, are known to have higher level of oxidative stress than non-transformed cells [28, 29]. Moreover, many therapeutic interventions further increase ROS production. Sublethal oxidative stress accelerates tumor progression and raises the risk of metastasis by increasing mutation rate, cell growth and blood supply signalling pathways [30]. The data presented here corroborate the antioxidant activity of HT in hypoxic but not in normoxic conditions. These results resemble the previous findings by Warleta et al. [13], who described that HT did not exert any antioxidant effect in MCF-7 cells cultured in standard conditions, but was antioxidant after inducing a ROS burst with H_2_O_2_. In tumors we can find two different types of hypoxia [31]: i) chronic hypoxia, adjacent to necrotic areas, due to a distance between the cells and the vasculature greater than the diffusion distance of oxygen (100–150 μm) [32]; and ii) acute hypoxia, caused by temporary obstruction or variable blood flow in tumor vessels [33]. The latter involves reperfusion periods in which ROS are further increased. Consequently, our results support the notion that in tumors HT may be beneficial to minimize the ROS burst associated to hypoxia. A lower oxidative stress level in breast cancer patients receiving chemotherapy correlates with higher survival rates [34], and it has been recently described that the co-treatment with paclitaxel and HT decreases the oxidative stress without counteracting the effect of chemotherapy [14]. Therefore, the hypoxic environment may have exerted a crucial role in this beneficial effect of HT.

Mitochondria are one of the major sources of ROS production, and clinical studies have reported altered mitochondrial function in human cancers [29]. PGC-1α, a major regulator of mitochondrial function, oxygen consumption and oxidative phosphorylation [35], is known to be induced in hypoxic conditions [36]. The link between HT and PCG-1α expression has been scarcely analysed in the literature and even less in hypoxic conditions. In adipocytes and in excessive exercised-rats, HT supplementation has been reported to increase PGC-1α level and the activity and expression of mitochondrial electron complexes [37, 38]. Similarly, Signorile et al. [39] described that the repression of PCG-1α and mitochondrial oxidative phosphorylation in serum starved fibroblast was reversed by HT-treatment. However, those authors only analysed the effect of HT at the protein level. In the present study we evaluated the transcriptional and translational effects of HT on PGC-1α expression. At the mRNA level, no effect was observed with the lowest concentrations of HT, but strikingly high doses (100 and 200 μM) induced a sharp decrease in PGC-1α transcription in normoxic and hypoxic cells. Therefore, the transcriptional effect of HT PGC-1α does not seem to be modulated by the hypoxic environment. Interestingly, at the protein level results were different. In agreement with previous reports, our data reinforced the positive influence of HT on the expression of the PCG-1α. Nevertheless, this effect was abolished in a hypoxic environment, suggesting that the antioxidant action of HT in this situation should be achieved by other regulatory mechanisms. In this sense, we could hypothesize that an increased activity of the pre-existing PGC-1α protein, rather than an increased expression, may be involved in the antioxidant action of HT under hypoxic conditions.

The effect of PGC-1α is mediated by coactivation of transcription factors such as ERRα. ERRα is an orphan nuclear receptor with a demonstrated role in tumor biology, and particularly in breast cancer. In fact, a relation between elevated ERRα activity and a shorter disease-free survival was described in a genomic analysis of more than 800 breast tumors [5]. It has also been published that the downregulation of ERRα in MCF-7 cells inhibits cell proliferation and decreases the growth of xenografts [5, 40]. Nevertheless, our data indicated that ERRα hardly responds to HT treatment neither in normoxia nor in hypoxia, suggesting that the effects of HT cannot be attributed to changes in the level of this protein. The transcriptional activity of ERRα seems to be independent of ligand binding but is regulated by the expression and activity of its coregulators. SIRT3, a major mitochondrial NAD^+^-dependent deacetylase, which has been related to poor prognosis in breast cancer [41], is a target gene of ERRα/PGC-1α. The effect of HT on the expression of SIRT3, at the transcriptional and translational level, perfectly reproduced those of ERRα. Thus, even though this polyphenol does not seem to increase the expression of ERRα protein, the up-regulation of SIRT3 mRNA indicates a higher activity of the PGC-1α/ERRα pathway that again does not involve a change in SIRT3 protein. These inconsistent results may be attributed to the existence of post-transcriptional mechanisms of regulation and have also been reported recently in mice receiving a diet supplemented with HT [42]. In these animals, phosphorylation is one of the biological processes described to be modulated, and it has been previously published that HT-treatment significantly increases the activity of Akt [43]. Given that the transcriptional activity of ERRα is induced by this kinase [44], it could be speculated that HT may promote the increase in the mRNA of SIRT3 by activating ERRα, rather than by increasing ERRα protein. Non-coding RNAs and RNA-binding proteins are known to exert a crucial role in translation, and the modulatory role of HT on the expression of both of them has already been suggested [42, 45]. Besides, HT also regulates the expression of different translation factors in a cell dependent manner [42]. The effect of HT on all these molecules could hinder the translation of the components of the PGC-1α/ERRα pathway. Further studies evaluating the influence of HT on these regulatory molecules in both normoxic and hypoxic conditions would be necessary in order to unravel the complex transcriptional/translational modulation induced by this polyphenol.

Nrf2, also coactivated by PGC-1α, was initially described as an activator of the cytochrome oxidase subunit IV. However, today it is known to regulate the expression of hundreds of genes with ARE sequences. Although the effect of HT on Nrf2 at the protein level has already been published, the literature regarding a transcriptional regulation of HT on Nrf2 is scarce. Hao et al. [37] analysed the transcription of Nrf2 after 48 h of treatment with HT (0–50 μM) and, strikingly, they observed an enhanced mRNA level only at 1 μM, but the mechanisms involved in this effect remain to be elucidated. The data presented here showed that, in MCF-7 cells, HT induces a transcriptional up-regulation of Nrf2 at 200 μM, which involves a parallel protein increase. Furthermore, in contrast to the results observed in the analysis of PGC-1α, this regulatory role of HT was preserved in hypoxic conditions suggesting that Nrf2 exerts a crucial role in the response to HT. Nrf2 induces the expression of over a hundred protective genes that, among other functions, are involved in ROS scavenging and detoxification of carcinogens. To act as a transcription factor, Nrf2 must translocate to the nucleus where it will bind to the ARE of its target genes, inducing gene transcription. However, under basal conditions, Nrf2 is kept in the cytoplasm and targeted to degradation by its binding to a cysteine residue of the Kelch-like ECH-associated protein 1 (Keap1). In this sense, the induction of Nrf2 by HT has been attributed to: i) the phosphorylation of Nrf2 by kinases such as ERK and Akt [43], and ii) the alkylation of the cysteine residue involved in Nrf2/Keap binding by the ortho-quinone intermediate of HT [46]. These post-translational modifications will allow Keap-1 dissociation from Nrf2, which will then translocate to the nucleus and act as a transcription factor. GSTA2 and HO-1 are some of the antioxidant proteins induced by Nrf2. GSTA2 functions in the detoxification of electrophilic compounds and also exhibits glutathione peroxidase activity. HO-1 is the inducible isoform of HO in mammals, and catalyses the degradation of heme group into carbon monoxide and the antioxidant biliverdin/bilirubin with the parallel release of iron, which will be sequestered by ferritin. In the present study, the increase in Nrf2 protein observed in normoxic and hypoxic cells after treatment with HT 200 μM involved a higher transcription of both target genes, supporting the proposition that HT induces not only the expression, but also the activity of Nrf2. Nevertheless, this increased mRNA levels were not translated in proteins, with the exception of HO-1 whose expression was increased only in normoxia. These findings, together with those of PGC-1α, seem to suggest that the translational effects of HT are at least partially down-regulated in a hypoxic situation, and consequently the antioxidant effect of HT in hypoxic conditions can be hardly attributed to an increased protein expression of antioxidant enzymes.

Most of the studies regarding HT suggest the crucial role of Nrf2 in the effect of this polyphenol and, as shown above, in MCF-7 cells HT promotes the expression of Nrf2 both in normoxia and in hypoxia. However, antioxidant enzymes can also be increased by Nrf2-independent mechanisms. In fact, by silencing Nrf2, we have demonstrated that although GSTA2 is exclusively dependent on Nrf2, other mechanisms seem be involved in the transcription of HO-1. The Nrf2 independent induction of HO-1 has been previously reported in the literature. In this sense, the natural xanthone gartanin is known to induce HO-1 independently of Nrf2 [47], and nitro-linoleic acid was described to promote HO-1 expression in Nrf2 deficient cells through cAMP, AP-1 and E-box response element interactions [48]. Moreover, in muscle atrophy, HO-1 increase was dependent on forkhead box O1 (FOXO1) but not on Nrf2 [49]. Therefore, our results evidence the existence of Nrf2-independent mechanisms also involved in the effect of HT.

## Conclusions

**Fig 7:**
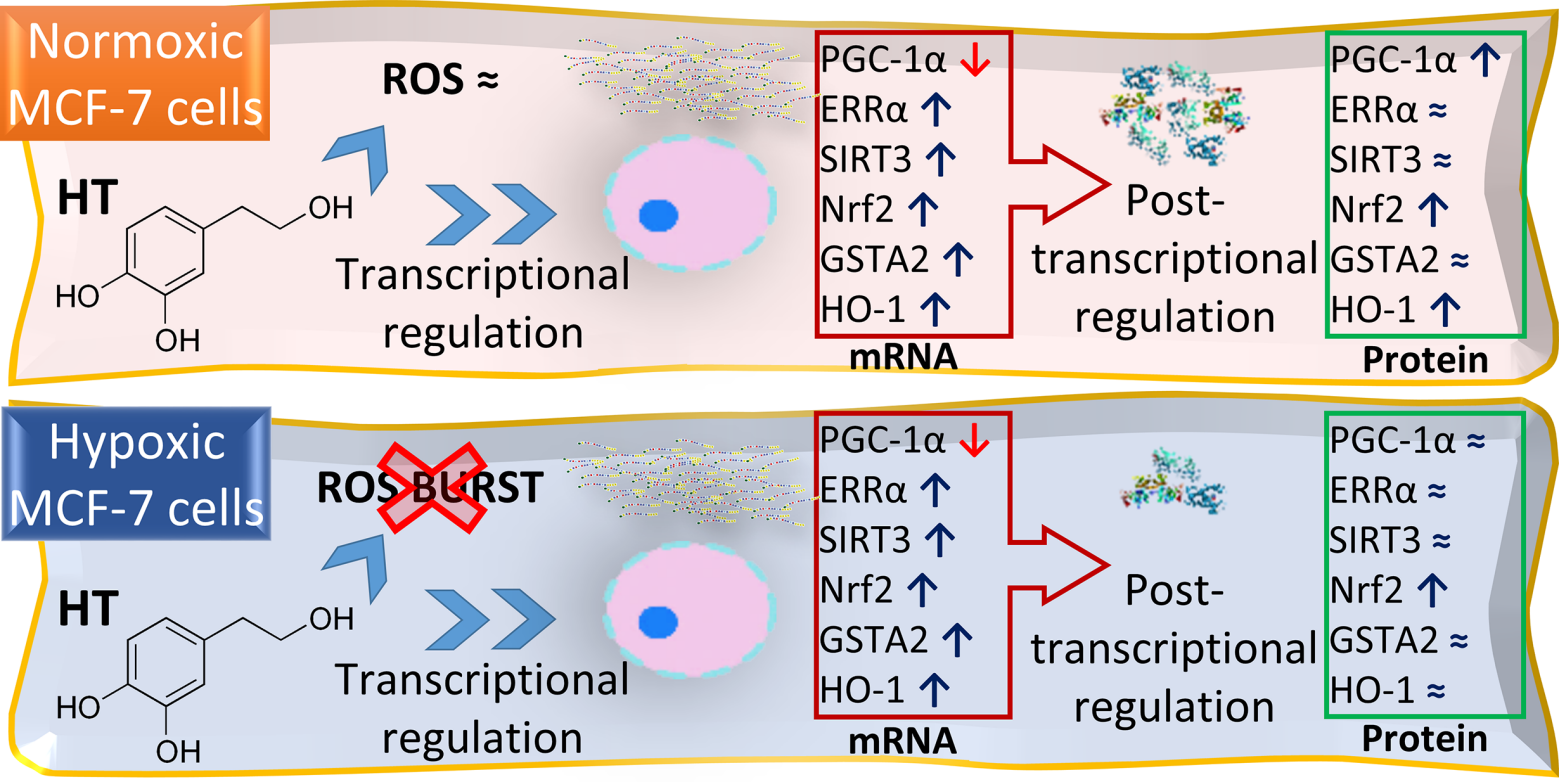
Comparative effect of HT on the oxidative response of MCF-7 cells in normoxic and hypoxic conditions. HT-treatment dampens the hypoxia-associated oxidative stress increase. The transcriptional regulation exerted by this polyphenol on the PGC-1α/ERRα and PGC-1α/Nrf2 pathways is not affected by O_2_ tension. However, hypoxia attenuates its translational effect, maybe due to the induction of posttranscriptional mechanisms of regulation by HT.

In conclusion, the data presented here and summarized in Fig 7 suggest that the antioxidant action of HT is particularly effective in hypoxic conditions. This polyphenol modulates the transcription and translation of different proteins of the PGC-1α/ERRα and PGC-1α/ Nrf2 pathways, typically involved in the antioxidant response. While the transcriptional effects of HT are similar in normoxic and hypoxic conditions, its translational action is less prominent and partially attenuated in a hypoxic environment. Therefore: i) the direct effect of this polyphenol must be the main responsible for its antioxidant action in hypoxia; ii) the discordance between the mRNA and protein results points to the importance of assessing the regulatory role of HT at both levels to get a complete picture of HT effects, and iii) the hypoxic environment of tumor cells should not be diminished when analyzing the effect of this or other bioactive compounds.

## Supporting information

S1 File Cytotoxic effect of HT in normoxic and hypoxic MCF-7 cells. (XLSX)

S2 File Effect of HT on the oxidative stress level in normoxic and hypoxic MCF-7 cells. (XLSX)

S3 File Effect of HT treatment on the expression of PGC-1α. (XLSX)

S4 File Effect of HT treatment on the expression of ERRα and SIRT3. (XLSX)

S5 File Effect of HT treatment on Nrf2 expression and activity. (XLSX)

S6 File Effect of Nrf2 silencing on the mRNA levels of GSTA2 and HO-1 in normoxic and hypoxic MCF-7 cells after treatment with HT 200 μM. (XLSX)
